# Reduction in peripheral vascular resistance predicts improvement in insulin clearance following weight loss

**DOI:** 10.1186/s12933-015-0276-2

**Published:** 2015-08-22

**Authors:** Nora E. Straznicky, Mariee T. Grima, Carolina I. Sari, Elisabeth A. Lambert, Sarah E. Phillips, Nina Eikelis, Daisuke Kobayashi, Dagmara Hering, Justin A. Mariani, John B. Dixon, Paul J. Nestel, Sofie Karapanagiotidis, Markus P. Schlaich, Gavin W. Lambert

**Affiliations:** Laboratory of Human Neurotransmitters, Baker IDI Heart and Diabetes Institute, P.O. Box 6492, St Kilda Road Central, Melbourne, VIC 8008 Australia; Laboratory of Neurovascular Hypertension and Kidney Disease, Baker IDI Heart and Diabetes Institute, Melbourne, VIC Australia; Heart Failure Research Group, Baker IDI Heart and Diabetes Institute, Melbourne, VIC Australia; Laboratory of Cardiovascular Nutrition, Baker IDI Heart and Diabetes Institute, Melbourne, VIC Australia; Alfred Baker Medical Unit, Baker IDI Heart and Diabetes Institute, Melbourne, VIC Australia; Faculty of Medicine, Nursing and Health Sciences, Monash University, Melbourne, VIC Australia; Department of Physiology, Monash University, Melbourne, VIC Australia; Department of Primary Health Care, Monash University, Melbourne, VIC Australia; Department of Physiology, University of Melbourne, Melbourne, VIC Australia

**Keywords:** Insulin clearance, Weight loss, Vascular resistance, Obesity, Norepinephrine kinetics

## Abstract

**Background:**

The hyperinsulinemia of obesity is a function of both increased pancreatic insulin secretion and decreased insulin clearance, and contributes to cardiovascular risk. Whilst weight loss is known to enhance insulin clearance, there is a paucity of data concerning the underlying mechanisms. This study was conducted to examine the inter-relationships between changes in sympathetic nervous system (SNS) activity, vascular function and insulin clearance during a weight loss program.

**Methods:**

Seventeen non-smoking, un-medicated individuals aged 55 ± 1 years (mean ± SEM), body mass index (BMI) 33.9 ± 1.7 kg/m^2^, underwent a 4-month hypocaloric diet (HCD), using a modified Dietary Approaches to Stop Hypertension diet, whilst seventeen age- and BMI-matched subjects acted as controls. Insulin sensitivity and insulin clearance were assessed via euglycemic hyperinsulinemic clamp (exogenous insulin clearance); hepatic insulin extraction was calculated as fasting C-peptide to insulin ratio (endogenous insulin clearance); SNS activity was quantified by microneurographic nerve recordings of muscle sympathetic nerve activity (MSNA) and whole-body norepinephrine kinetics; and vascular function by calf venous occlusion plethysmography and finger arterial tonometry.

**Results:**

Weight loss averaged −8.3 ± 0.6 % of body weight in the HCD group and was accompanied by increased clamp-derived glucose utilization (by 20 ± 9 %, P = 0.04) and exogenous insulin clearance (by 12 ± 5 %, P = 0.02). Hepatic insulin extraction increased from 6.3 ± 0.8 to 7.1 ± 0.9 (P = 0.09). Arterial norepinephrine concentration decreased by −12 ± 5 %, whole-body norepinephrine spillover rate by −14 ± 8 %, and MSNA by −9 ± 5 bursts per 100 heartbeats in the HCD group (P all >0.05 versus control group). Step-wise regression analysis revealed a bidirectional relationship between enhanced exogenous insulin clearance post weight loss and reduction in calf vascular resistance (r = −0.63, P = 0.01) which explained 40 % of the variance. Increase in hepatic insulin extraction was predicted by enhanced finger reactive hyperaemic response (P = 0.006) and improvement in oral glucose tolerance (P = 0.002) which together explained 64 % of the variance.

**Conclusions:**

Insulin clearance is independently and reciprocally associated with changes in vascular function during weight loss intervention.

Trial registration ClinicalTrials.gov: NCT01771042 and NCT00408850

## Background

Hyperinsulinemia is a common characteristic of obesity and has been prospectively linked to the development of cardiovascular morbidity and mortality [1, 2]. The elevation in peripheral insulin concentration under both fasting and stimulated conditions has been attributed to the combined effects of increased pancreatic insulin secretion and reduced insulin clearance, physiological processes that are a function of the prevailing level of insulin resistance [3–5]. Indeed, longitudinal studies using a fat fed canine model [6] and intentional weight gain in healthy humans [7] indicate that reduced insulin clearance is an early adaptive phenomenon that may operate to preserve pancreatic β-cell function from excessive demand in the insulin resistant state. Within obese populations, upper body fat distribution, increased liver fat content, hypertension, hyperglycemia and dyslipidemia (known collectively as the metabolic syndrome), have been shown to confer lower insulin clearance [5, 8–12]. Despite its importance in the metabolism of insulin and its potential role in the aetiology of cardiovascular disease and diabetes [13], relatively little is known concerning the mechanisms responsible for impaired insulin clearance in obesity and for the recognized benefits of weight loss in this regard [14, 15].

The liver and the kidneys are the principle sites of insulin clearance from the circulation, accounting for approximately 60 and 25 % respectively of total clearance at physiological insulin levels [16], although other insulin sensitive tissues such as muscle and adipose tissue also participate to a lesser extent. Insulin uptake and degradation involves several steps, including binding to the insulin membrane receptor, cellular internalization and enzymatic degradation [17]. Our group has recently demonstrated inverse associations between resting sympathetic nervous system (SNS) activity, quantified as arterial norepinephrine concentration and whole-body norepinephrine spillover rate, peripheral arterial stiffness and insulin clearance in obese individuals with metabolic syndrome [18]. This concurs with an earlier study which showed that acute infusion of the α_1_-adrenergic agonist phenylephrine during hyperinsulinemic euglycemic clamp, decreased insulin clearance by 20 % in healthy volunteers [19]. Putative mechanisms by which catecholamine stimulation may adversely impact on insulin clearance include hemodynamic alterations such as enhanced vasoconstrictor tone and microvascular rarefaction that promote insulin resistance [20, 21], reduction in hepatic or renal blood flow [22, 23] and modulation of insulin receptor numbers and/or insulin binding affinity [24, 25]. Whilst the reciprocal interplay between insulin resistance, hyperinsulinemia and SNS activation has been long appreciated [21, 26], there is a paucity of knowledge regarding their interrelationship to insulin clearance, particularly in the context of weight loss which is known to elicit both sympathoinhibition and enhanced insulin sensitivity.

The aims of the present study were (1) to examine the effects of weight loss induced by hypocaloric diet (HCD) on insulin clearance, SNS activity and vascular function and (2) to delineate the pathophysiological drivers of improvement in insulin clearance following lifestyle intervention. We hypothesized that reduction in SNS activity would predict improvement in insulin clearance.

## Methods

### Subjects

Seventeen overweight and obese subjects were recruited through newspaper advertisements to participate in a weight loss trial (NCT01771042). They were compared with age- and BMI-matched controls (n = 17) who had been studied previously in our laboratory (NCT00408850). Entry criteria and study methodology were identical in the two studies. Subjects were non-smokers, aged 45–65 years, with a stable body weight (±1 kg) in the previous 6 months and were not taking medications that could affect study parameters (e.g. oral hypoglycemics, cholesterol-lowering, anti-hypertensive, anti-depressant and hormone replacement therapies). Females were post-menopausal. Exclusion criteria comprised history of cardiovascular, cerebrovascular, renal, liver, or thyroid diseases and use of continuous positive airway pressure treatment. Screening tests included physical examination, electrocardiogram, biochemical tests, medical and dietary history, and measurement of supine resting blood pressure as the average of five readings after 5 min rest using a Dinamap monitor (Model 1846SX, Critikon Inc, Tampa, FL, USA). Clinical investigations were performed at baseline and after 16-weeks HCD or no treatment (controls). The project was approved by the Alfred Hospital Human Research Ethics Committee and written informed consent was obtained from each participant.

### Dietary intervention

Habitual dietary intake was assessed by 4-day prospective diet records using Australian food composition tables (Foodworks 7 Professional, Xyris Software, Kenmore Hills Qld 4069). Subjects were prescribed HCD at 25 % energy deficit using a modified Dietary Approach to Stop Hypertension (DASH) eating plan [27], with higher protein and lower carbohydrate content (25 % protein, 30 % fat and 45 % carbohydrate) to maximize fat loss [28]. The DASH eating pattern has an emphasis on fruits and vegetables, low-fat dairy, wholegrain cereals, legumes and nuts, lean meats, poultry and fish. Randomized controlled trials have established that this dietary pattern lowers blood pressure and lipid levels and favourably influences metabolic syndrome components when used as the background diet during weight reduction [27, 29, 30]. Subjects were provided with written dietary instructions, 14-day menu plans and recipes, and prepared food in their home environment. They attended fortnightly for body weight measurement and dietary counselling with the study dietician. With regards to exercise they were instructed to walk briskly for 30 min, at least 5 days per week, in accordance with the American Diabetes Association Position Statement [31] and this was monitored by 7-day pedometry. Control subjects were told to maintain their usual eating and exercise habits. Dietary intake was monitored by prospective 4-days diet records and 24-h urinalysis (collected on the day prior to SNS tests) to quantify sodium, potassium, urea and creatinine excretion. Clinical testing was performed on two separate mornings a week apart. Subjects attended at 8 am after an overnight fast and having abstained from alcohol and heavy exercise for 36 h and caffeine for 18 h. All experiments were performed in the supine position in a temperature controlled (22 °C) research room.

### Glucose metabolism and insulin clearance

Participants underwent a standard 75-g oral glucose tolerance test (OGTT, Glucaid, Fronine PTY, LTD, Taren Point, NSW 2229, Australia) with 30-min blood sampling. The Matsuda insulin sensitivity index (ISI) was calculated as a measure of insulin sensitivity to endogenously produced insulin [32]. On a separate day, the euglycemic hyperinsulinemic clamp was initiated by an intravenous bolus injection of insulin (9 mU/kg; Actrapid 100 IU/ml Novo Nordisk, Gentofte, Denmark), followed by a constant infusion rate of 40 mU m^2^ min^−1^. Arterialized blood glucose was clamped at 5.0 mmol/L by the variable infusion of 25 % glucose (Baxter, Toongabbie, Australia) and measured every 5-min using an ABL8XX glucose auto-analyzer (Radiometer, Copenhagen, Denmark). The mean glucose infusion rate between 90 and 120 min was used to calculate whole-body glucose utilization [M value, expressed as mg per min and mg per kg fat free mass (FFM) per min] and adjusted for steady-state insulin (M/I value). Fasting and steady-state C-peptide concentrations were measured to verify suppression of endogenous insulin secretion.

Whole-body exogenous insulin clearance was calculated by dividing the insulin infusion rate by the mean steady-state plasma insulin concentration (average of measurements at 90, 100, 110 and 120 min) with correction for changes in residual endogenous insulin secretion according to the formula [33]:

$${\text{Insulin clearance }}\left( {{\text{ml}}/{\text{m}}^{ 2} /{ \hbox{min} }} \right) = \frac{{{\text{Insulin infusion rate }}\left( {{\text{mU}}/{\text{m}}^{ 2} /{ \hbox{min} }} \right)}}{{\left[ {{\text{SS}}_{\text{i}} {-} \, \left( {{\text{B}}_{\text{i}} \times {\text{ SS}}_{\text{CP}} /{\text{B}}_{\text{CP}} } \right)} \right]}} \times 1000$$

where SS_i_ represents steady-state plasma insulin levels during the clamp (mU/L), B_i_ represents basal or fasting plasma insulin (mU/L) and SS_CP_ and B_CP_ are steady-state and basal C-peptide concentrations (pmol/L) respectively. Hepatic insulin extraction (endogenous insulin clearance) was calculated as the ratio of fasting C-peptide to fasting insulin concentration (both values expressed as pmol/L). This is based on the assumption that C-peptide is secreted equimolarly with insulin but is not subjected to hepatic first-pass metabolism.

Body weight was measured in light indoor clothes without shoes using a digital scale. Body composition was determined by whole-body dual-energy X-ray absorptiometry (DEXA, GE-LUNAR Prodigy Advance PA+130510, GE Medical Systems, Lunar, Madison, W1, USA).

### Norepinephrine kinetics

Fasting norepinephrine kinetics were determined on the same morning and prior to the OGTT. After cannulation of an antecubital vein and the brachial artery, fasting venous blood samples were obtained for biochemical measurements. Radioisotope dilution methodology was then used to quantify the rate at which norepinephrine released from sympathetic nerve endings enters the central plasma compartment (whole-body norepinephrine ‘spillover’ rate) [34]. This technique involves the intravenous infusion of [^3^H]-norepinephrine and measurement of norepinephrine specific activity and endogenous norepinephrine in arterial blood, sampled from the brachial artery, under steady-state conditions. After a priming bolus of 1.81 μCi of 1-[ring-2,5,6-^3^H]-norepinephrine (Perkin-Elmer, Waltham, MA, USA; specific activity, 10–30μCi/mmol), an infusion was commenced at 0.18 μCi min^−1^, with arterial blood sampling 30 min after infusion commencement. The following calculations were performed:

$${\text{Norepinephrine clearance }}\left( {{\text{L}}/{ \hbox{min} }} \right) = \frac{{[^{ 3} {\text{H}}] - {\text{norepinephrine infusion rate }}\left( {{\text{dpm}}/{ \hbox{min} }} \right)}}{{[^{ 3} {\text{H}}] - {\text{norepinephrine plasma concentration }}\left( {{\text{dpm}}/{\text{ml}}} \right) \times 1000}}$$

$${\text{Norepinephrine spillover }}\left( {{\text{ng}}/{ \hbox{min} }} \right) = \frac{{{\text{plasma norepinephrine }}\left( {{\text{pg}}/{\text{ml}}} \right) \, \times {\text{ clearance }}\left( {{\text{ml}}/{ \hbox{min} }} \right)}}{ 1000}$$

After completion of arterial blood sampling, subjects underwent an OGTT as described above. It is of note that high specific activity [^3^H]-norepinephrine has no physiological effects at the doses used [34].

### Muscle sympathetic nerve activity

Resting multi-unit muscle sympathetic nerve activity (MSNA) activity was measured via a tungsten microelectrode inserted into a muscle nerve fascicle of the right peroneal nerve at the fibular head. Once an acceptable nerve-recording site was obtained via visual and acoustic identification of spontaneous sympathetic bursts, resting measurements were recorded over 15 min. The nerve signal was amplified (×50,000), filtered (bandpass, 700–2000 Hz) and integrated. Continuous electrocardiographic and MSNA recordings were digitised with a sampling frequency of 1000 Hz (PowerLab recording system, model ML785/8SP, AD Instruments). MSNA was manually analysed and expressed as burst frequency (bursts/min) and burst incidence (bursts/100 heartbeats), averaged over 15 min.

### Cardiovascular parameters

Resting cardiac baroreflex sensitivity was estimated using the sequence method. This procedure identifies the spontaneous sequences of three or more consecutive heartbeats during which intra-arterial systolic blood pressure increased and cardiac interval lengthened (type 1 sequences) or systolic blood pressure decreased and cardiac interval shortened (type 2 sequences), with a lag of one beat. The slope of the regression line between cardiac interval and intra-arterial systolic blood pressure values was calculated for each validated sequence (when r > 0.85). Individual slopes were averaged over a 15 min recording period.

Cardiac output was quantified by transthoracic Doppler echocardiography (Vivid 7 GE Vingmed, GE Healthcare) from the left ventricular outflow tract cross-sectional diameter and the velocity time integral measurements.

Calf blood flow measurements were obtained in the left leg by venous occlusion plethysmography (D.E.Hokansen, Bellevue, WA, USA). Strain gauges were attached around the thickest part of the calf and the measured leg was supported 10 cm above heart level to empty the venous system. Twelve consecutive readings were averaged and expressed as mL per min per 100 g of tissue. Calf vascular resistance was calculated as mean arterial pressure (MAP) divided by calf blood flow. Measurements were made in the fasting state and every 30 min during OGTT to quantify the vasodilatory response to endogenous insulin.

Endothelial function was evaluated non-invasively in the index finger of the non-dominant hand by the EndoPAT 2000 device (Itamar Medical Ltd, Caesarea, Israel). A beat-to-beat plethysmographic recording of the arterial pulse-wave was obtained over 5 min of supine rest. Brachial arterial occlusion was then induced by inflating an upper arm cuff to supra-systolic pressure (60 mmHg above systolic pressure) for 5 min, and released to elicit reactive flow-mediated hyperaemia. Pulse amplitude responses to hyperaemia were calculated from the hyperaemic fingertip as the ratio of the post deflation pulse amplitude between 90 and 120 s to the baseline pulse amplitude and adjusted for the corresponding ratio in the contralateral control hand (PAT ratio) [35].

### Laboratory analyses

Plasma glucose and lipid profile were measured on a commercial analytical system (Architect C18000 analyzer, Abbott Laboratories, Illinois, USA). High sensitivity C-reactive protein (*hs*-CRP was quantified by immunoturbidimetric assay, insulin and leptin by radio-immuno assay (Linco Research, Inc, Missouri, USA), C-peptide by chemiluminescent immunoassay (ADVIA Centaur, Siemens Healthcare Diagnostics, Tarrytown, NY, USA) and non-esterified fatty acids (NEFA) by enzymatic colorimetric method (Wako Pure Chemical Industries, Ltd, Osaka, Japan). Plasma norepinephrine was assayed by high performance liquid chromatography with electrochemical detection, following extraction by alumina adsorption. [^3^H]-norepinephrine was assayed by liquid scintillation chromatography and corrected for loss during extraction using recoveries of internal standard. Intra-assay CVs in our laboratory are 1.3 % for norepinephrine and 2.3 % for [^3^H]-norepinephrine; inter-assay CVs are 3.8 and 4.5 %, respectively.

### Statistical analyses

Statistical analysis was performed using SigmaStat Version 3.5 (Systat Software Inc, Point Richmond, CA, USA). Data are presented as the mean ± SEM. Baseline parameters in the two study groups were compared by un-paired t-tests and Mann–Whitney rank sum test. Two-way repeat measures ANOVA was used to test for group effects, time effects and group × time interactions, with the Holm-Sidak test for multiple post hoc comparisons. Non-parametric data were log transformed. Post-intervention changes in study parameters (Δ) were compared by un-paired *t* test or Mann–Whitney test as appropriate. Sub-group analysis by baseline insulin status, was performed using a cut-point of insulin area under the curve during OGTT (AUC_0–120_) of 8000 mU/L per minute, to categorize subjects as hyperinsulinemic or normoinsulinemic [36]. Univariate associations between change in insulin clearance and other variables were assessed using Pearson’s correlations. Forward stepwise linear regression analysis, adjusted for age and change in body weight, was performed to identify the independent predictors of change in insulin clearance in the HCD group. Variables with P values <0.10 in univariate analyses were entered into the regression model. Statistical significance was accepted at the P < 0.05 (two-tailed) level.

## Results

### Subjects

Baseline demographic, clinical and dietary characteristics of study participants are presented in Table 1. Control and HCD groups were well matched for age, anthropometrics, glucose metabolism and blood pressure. Body weight and total body fat mass decreased by −8.3 ± 0.6 % (range −3.6 to −11.6 %) and −6.2 ± 0.7 kg respectively and plasma leptin by −8.5 ± 2.1 ng/mL in the HCD group (all P < 0.001 versus control group, Table 2). Based on 4-day diet records, energy deficit averaged −543 ± 100 kcal/day, carbohydrate and saturated fat intake decreased by −6.3 ± 1.4 % and −2.9 ± 0.8 % (−12 ± 3 g/day) of energy respectively, and protein intake increased by 7.5 ± 1.2 % of energy (all P ≤ 0.001). Urinary sodium excretion decreased by −42 ± 18 mmol/day in the HCD group and −5 ± 13 mmol/day in the control group (P = 0.11), whereas potassium and urea excretion were unchanged. Pedometry records showed an increment of 1419 ± 561 steps/day in the HCD (P = 0.03).

**Table 1:** Demographic, clinical and dietary variables of study participants

	Control (n = 17)	HCD (n = 17)	P
Age (years)	56 ± 1	55 ± 1	0.87
Gender (M/F)	10/7	7/10	0.49
Number of MetS components	2.7 ± 0.3	2.5 ± 0.3	0.68
Clinical parameters			
Body weight (kg)	97.8 ± 4.0	98.6 ± 4.1	0.89
Body mass index (kg/m^2^)	32.8 ± 0.9	33.9 ± 1.7	0.85
Waist circumference (cm)	106.1 ± 2.3	107.8 ± 2.8	0.63
Waist to hip ratio	0.91 ± 0.02	0.91 ± 0.02	0.96
Fasting glucose (mmol/L)	5.8 ± 0.1	6.1 ± 0.2	0.24
2-h glucose (mmol/L)	9.6 ± 0.7	10.4 ± 0.7	0.44
Fasting insulin (mU/L)	17.7 ± 1.6	18.0 ± 1.4	0.88
HOMA-IR	4.8 ± 0.4	4.9 ± 0.5	0.90
M (mg kg FFM min^−1^)	9.49 ± 0.82	10.24 ± 0.93	0.55
Insulin clearance (mL m^2^ min^−1^)	382 ± 20	352 ± 14	0.23
Triglycerides (mmol/L)	1.5 ± 0.1	1.7 ± 0.2	0.77
HDL-cholesterol (mmol/L)	1.29 ± 0.07	1.20 ± 0.06	0.44
LDL-cholesterol (mmol/L)	3.4 ± 0.1	3.9 ± 0.2	0.07
Clinic systolic BP (mmHg)	127 ± 4	121 ± 2	0.22
Clinic diastolic BP (mmHg)	72 ± 2	69 ± 2	0.27
Clinic heart rate (bpm)	62 ± 2	62 ± 2	0.92
7-day pedometry (steps per day)	8021 ± 724	8824 ± 1100	0.68
Habitual dietary intake			
Energy (kcal/day)	2081 ± 158	2067 ± 86	0.69
Fat (% energy)	34 ± 2	36 ± 1	0.32
Saturated fat (% energy)	14 ± 1	15 ± 1	0.46
Protein (% energy)	19 ± 1	18 ± 1	0.83
Carbohydrate (% energy)	44 ± 2	41 ± 2	0.28
Alcohol (% energy)	2 ± 1	2 ± 1	0.74
24-h urinary sodium (mmol/day)	155 ± 20	157 ± 14	0.58

*BP* blood pressure, *HOMA-IR* homeostasis model assessment of insulin resistance, *M* steady state glucose utilization during euglycemic hyperinsulinemic clamp (mg per kg fat free mass per minute), *MetS* metabolic syndrome components defined as per harmonized definition

**Table 2:** Anthropometric, metabolic and cardiovascular parameters at baseline and post interventions

Variables	Control		HCD		Group × time interaction (P)
	Baseline	Week 16	Baseline	Week 16	
Anthropometric					
Body weight (kg)	97.8 ± 4.0	98.2 ± 4.2	98.6 ± 4.1	90.4 ± 4.0***	<0.001
Δ		0.4 ± 0.4		−8.1 ± 0.6^‡^	
Body mass index (kg/m^2^)	32.8 ± 0.9	32.9 ± 1.0	33.9 ± 1.7	31.2 ± 1.7***	<0.001
Δ		0.2 ± 0.2		−2.7 ± 0.2^‡^	
Waist circumference (cm)	106.1 ± 2.3	106.5 ± 2.2	107.8 ± 2.8	100.3 ± 2.8***	<0.001
Δ		0.4 ± 0.4		−7.5 ± 0.9^‡^	
Waist to hip ratio	0.91 ± 0.02	0.92 ± 0.02	0.91 ± 0.02	0.89 ± 0.01***	0.001
Δ		0.0 ± 0.0		−0.03 ± 0.01^‡^	
Total body fat mass (kg)	38.0 ± 2.9	38.0 ± 3.0	42.0 ± 2.7	35.8 ± 3.1***	<0.001
Δ		0.1 ± 0.3		−6.2 ± 0.7^‡^	
Metabolic and biochemical					
Fasting glucose (mmol/L)	5.8 ± 0.1	5.9 ± 0.1	6.1 ± 0.2	5.8 ± 0.1**	0.03
Δ		0.0 ± 0.1		−0.3 ± 0.1^†^	
Fasting insulin (mU/L)	18.8 ± 1.7	20.4 ± 1.6*	18.0 ± 1.4	13.8 ± 1.2***^‡^	<0.001
Δ		1.7 ± 0.9		−4.2 ± 0.7^‡^	
Triglycerides (mmol/L)	1.5 ± 0.1	1.4 ± 0.1	1.7 ± 0.2	1.1 ± 0.1***	0.02
Δ		−0.1 ± 0.1		−0.6 ± 0.2^†^	
HDL-cholesterol (mmol/L)	1.29 ± 0.07	1.20 ± 0.06*	1.21 ± 0.05	1.25 ± 0.07	0.02
Δ		−0.09 ± 0.04		0.04 ± 0.03^†^	
LDL-cholesterol (mmol/L)	3.4 ± 0.1	3.4 ± 0.2	3.8 ± 0.2	3.5 ± 0.2**	0.02
Δ		0.0 ± 0.1		−0.3 ± 0.1^†^	
NEFA (mEq/L)	0.53 ± 0.04	0.51 ± 0.04	0.57 ± 0.05	0.55 ± 0.04	0.93
Δ		−0.02 ± 0.03		−0.02 ± 0.04	
Leptin (ng/mL)	18.7 ± 4.6	19.1 ± 3.6	21.6 ± 4.7	13.1 ± 3.4***	<0.001
Δ		0.4 ± 1.6		−8.5 ± 2.1^‡^	
High sensitivity CRP (mg/L)	3.3 ± 0.8	3.5 ± 0.9	3.2 ± 0.6	2.2 ± 0.4*	0.12
Δ		0.2 ± 0.6		−0.9 ± 0.3^†^	
Creatinine clearance (mL/min)	150 ± 11	147 ± 9	140 ± 10	118 ± 7**^†^	0.06
Δ		−3 ± 6		−22 ± 10	
Cardiovascular					
Clinic MAP (mmHg)	92 ± 3	95 ± 3	89 ± 2	86 ± 2*	0.007
Δ		3 ± 2		−3 ± 1^‡^	
Clinic heart rate (bpm)	62 ± 2	61 ± 2	62 ± 2	59 ± 2*	0.41
Δ		−2 ± 1		−3 ± 1	
Cardiac BRS (ms/mmHg)	15.7 ± 2.2	15.7 ± 1.9	18.0 ± 2.6	23.7 ± 2.9*^†^	0.16
Δ		0.0 ± 2.2		5.8 ± 2.3	
Calf vascular resistance (units)	53.0 ± 6.7	57.4 ± 4.4	51.3 ± 4.9	47.1 ± 3.3	0.18
Δ		4.4 ± 4.6		−4.2 ± 3.9	
Finger reactive hyperaemic index	2.35 ± 0.15	2.42 ± 0.16	2.38 ± 0.19	2.26 ± 0.15	0.44
Δ		0.07 ± 0.13		−0.12 ± 0.20	
PAT ratio	0.68 ± 0.09	0.67 ± 0.09	0.67 ± 0.10	0.62 ± 0.09	0.77
Δ		−0.01 ± 0.10		−0.05 ± 0.11	
Cardiac output (L/min)	5.3 ± 0.3	5.6 ± 0.4	5.5 ± 0.4	5.4 ± 0.4	0.30
Δ		0.3 ± 0.2		−0.1 ± 0.2	

*BRS* baroreflex sensitivity, *CRP* C-reactive protein, *MAP* mean arterial pressure, *NEFA* non-esterified fatty acids, *PAT* peripheral arterial tonometry

* P < 0.05, ** P < 0.01 and *** P < 0.001 versus Baseline; ^†^P < 0.05 and ^‡^P ≤ 0.01 versus Control Group

Cholesterol profile improved significantly after weight loss: plasma LDL-cholesterol and triglyceride concentrations fell, whilst HDL-cholesterol increased compared to control subjects (all P < 0.05, Table 2). The inflammatory marker *hs*-CRP decreased significantly only in the HCD group.

### Glucose metabolism and insulin clearance

**Fig. 1:**
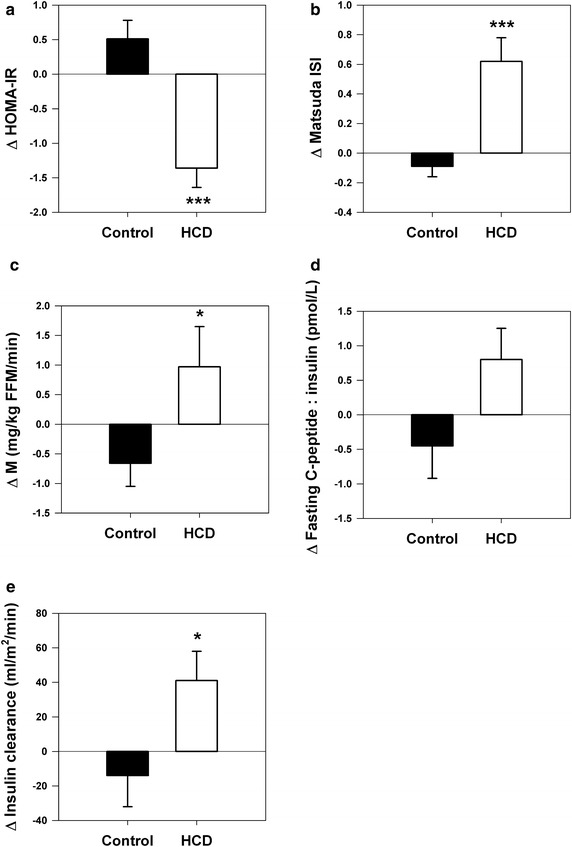
Changes in insulin sensitivity and insulin clearance in control and hypocaloric diet (HCD) groups (week 16 minus baseline measurements). **a** Homeostasis model assessment of insulin resistance (HOMA-IR): group × time interaction, P < 0.001. **b** Matsuda insulin sensitivity index (ISI): group × time interaction, P < 0.001. **c** Steady state glucose utilization during euglycemic clamp (M): group × time interaction, P = 0.02. **d** Fasting C-peptide to insulin ratio: group × time interaction, P = 0.06. **e** Insulin clearance: group × time interaction, P = 0.03. *P < 0.05 and ***P < 0.001 versus control group

Fasting glucose and insulin, and the glucose and insulin areas under the curve between time 0 and 120 min (AUC_0–120_) during the OGTT, decreased significantly in the HCD group (P all ≤0.01) and there was a commensurate reduction in HOMA-IR and an increase in Matsuda ISI (P both <0.001)(Fig. 1a, b). Clamp derived glucose utilization increased by an average of 20 ± 9 % in the HCD group (P = 0.02 versus control group) (Fig. 1c). Fasting C-peptide to insulin ratio increased from 6.3 ± 0.8 at baseline to 7.1 ± 0.9 post-weight loss in the HCD group (P = 0.09) (Fig. 1d). Exogenous insulin clearance increased from 352 ± 14 to 393 ± 21 mL/m^2^/min (P = 0.02) (Fig. 1e), representing and average increment of 12 ± 5 %. There were no changes in glucose or insulin parameters in the control group.

### Sympathetic nervous system activity

**Fig. 2:**
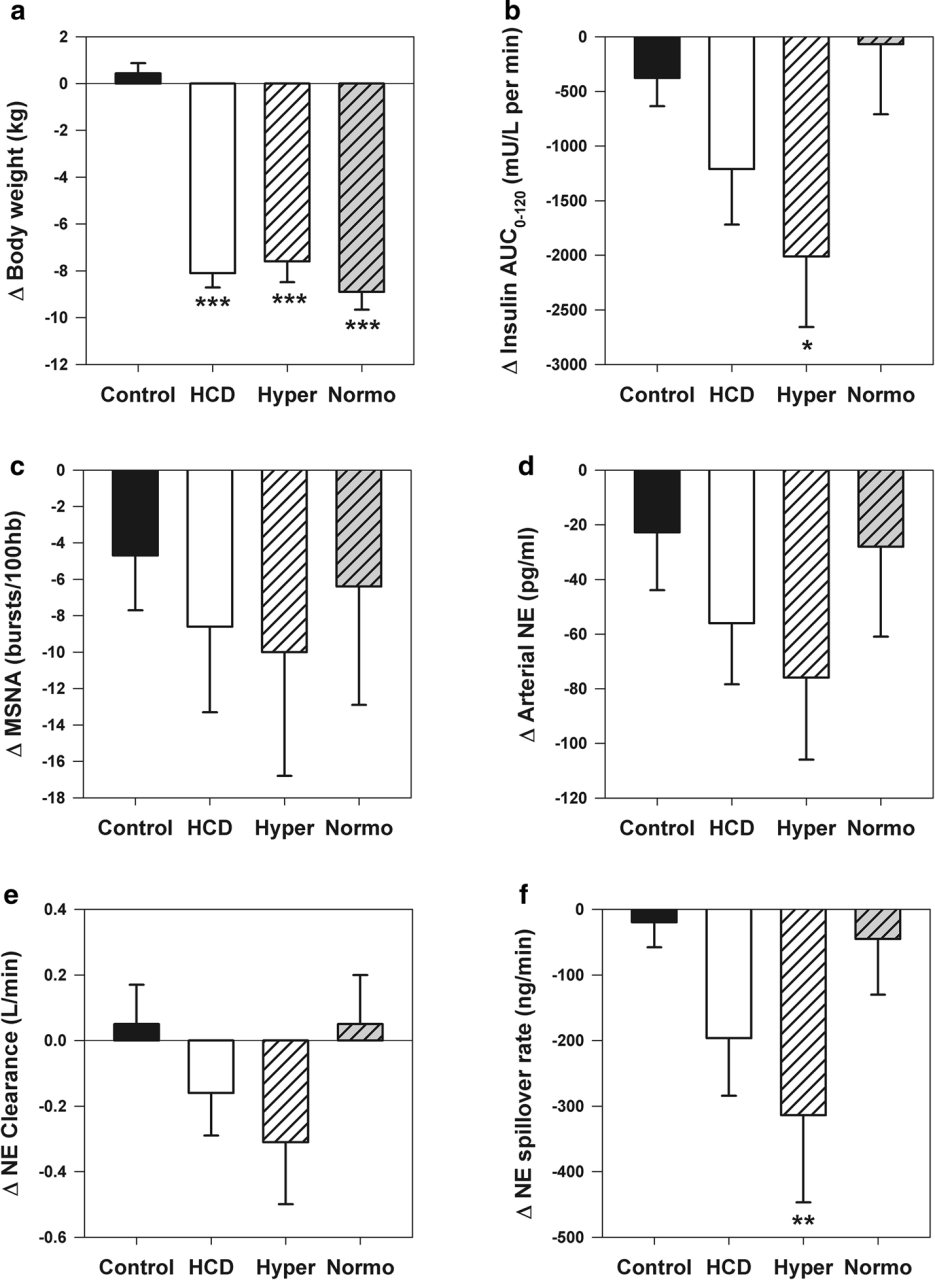
Change in sympathetic nervous system parameters in relation to change in body weight and insulin status in control and hypocaloric diet (HCD) groups (week 16 minus baseline measurements). HCD sub-groups are indicated by the *hashed bars* and include hyperinsulinemic (Hyper, n = 10) and normo-insulinemic (Normo, n = 7) subjects. **a** Body weight. **b** Insulin area under the curve during oral glucose tolerance test (AUC_0–120_). **c** Muscle sympathetic nerve activity (MSNA) burst incidence. **d** Arterial plasma norepinephrine (NE) concentration. **e** Norepinephrine plasma clearance. **f** Whole-body norepinephrine spillover rate. *P < 0.05, **P < 0.01 and ***P < 0.001 versus control group

Figure 2 illustrates changes in MSNA burst incidence and norepinephrine kinetics in relation to changes in body weight and insulin status in the study groups. Arterial norepinephrine concentration and calculated whole-body norepinephrine spillover rate decreased by −12 ± 5 % and −14 ± 8 % respectively in the HCD group, which did not differ significantly from changes in the control group. Satisfactory paired MSNA measurements were obtained in 10 control and 13 HCD subjects. MSNA decreased by −7 ± 3 bursts/min and −9 ± 5 bursts per 100 hb in the HCD, but this did not differ significantly from changes in the control group. Sub-group analyses by baseline insulin status (hyperinsulinemic: insulin AUC_0–120_ 10,394 ± 694 mU/L per min, n = 10; normoinsulinemic: insulin AUC_0–120_ 5469 ± 501 mu/L per min, n = 7), showed that significant sympathoinhibition (reduction in whole-body norepinephrine spillover rate) was only attained in hyperinsulinemic subjects following HCD, despite similar weight loss in the two sub-groups (Fig. 2f).

### Cardiovascular parameters

**Fig. 3:**
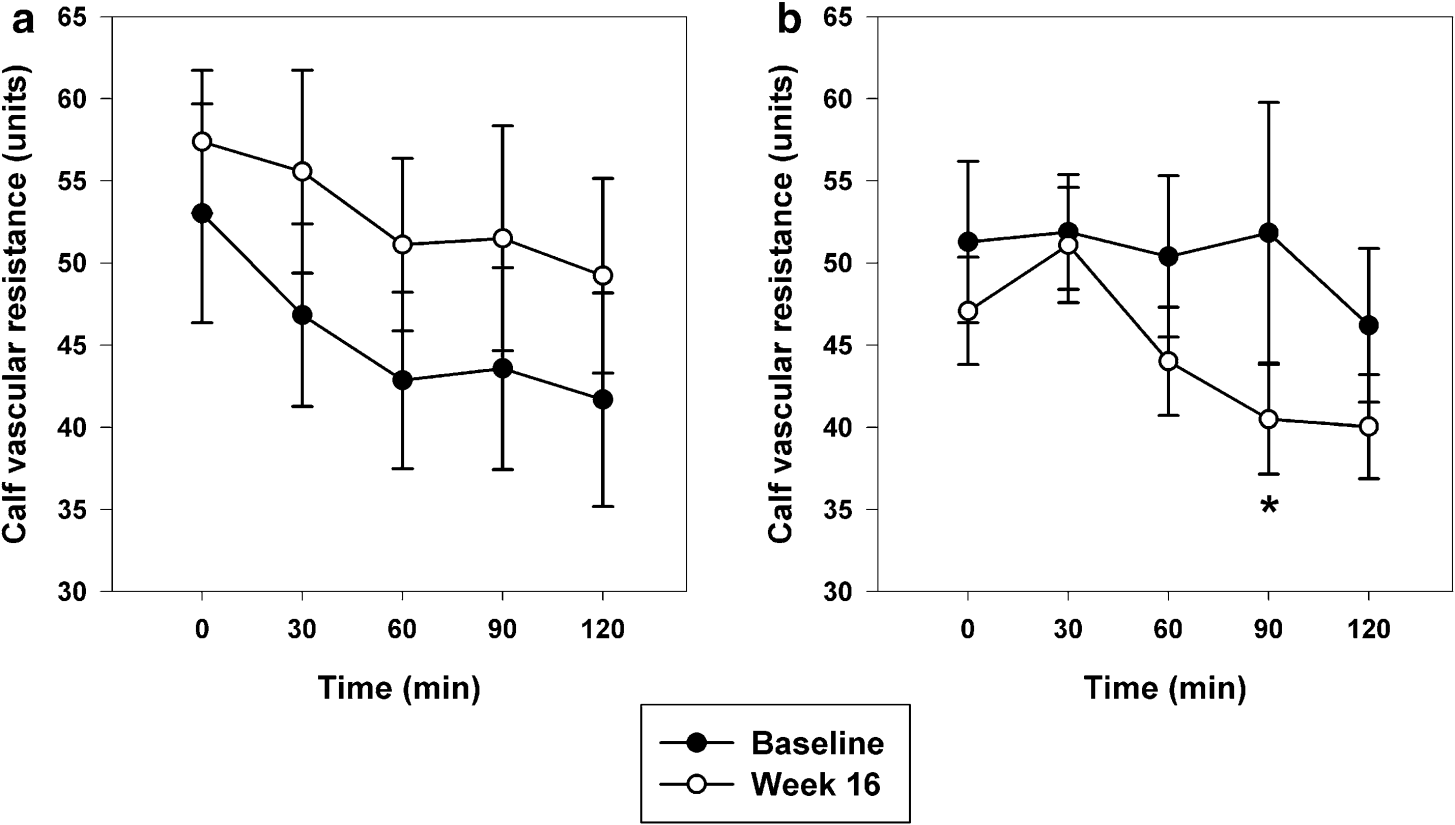
Calf vascular resistance during 75-g oral glucose tolerance test in **a** control and **b** HCD groups. *P < 0.05 versus baseline. Change in the area under the curve (AUC_0–120_) averaged 927 ± 555 units per min and −712 ± 526 units per min in control and HCD groups, respectively (P = 0.045)

Cardiovascular parameters are presented in Table 2. Clinic MAP was reduced and spontaneous cardiac baroreflex sensitivity was enhanced following weight loss (P both <0.05 versus control group). Cardiac output, finger reactive hyperaemic response and PAT ratio were not significantly altered with weight loss. Fasting calf vascular resistance decreased non-significantly after weight loss (P = 0.17 versus control) and there was a greater vasodilatory response to endogenous insulin during the OGTT in HCD, but not the control group (Fig. 3). Baseline insulin status did not modify changes in cardiovascular parameters following HCD.

### Correlation and regression analyses

**Fig. 4:**
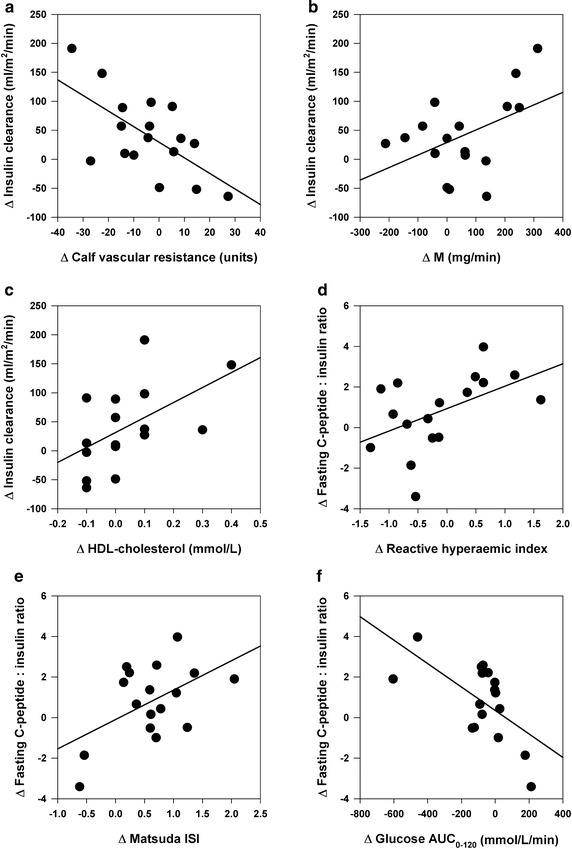
Univariate correlates of change in whole-body insulin clearance and hepatic insulin extraction (fasting C-peptide to insulin ratio) in the HCD group (n = 17). **a** Change in fasting calf vascular resistance (r = −0.63, 0.007). **b** Change in steady state glucose utilization during euglycemic hyperinsulinemic clamp (M) (r = 0.45, P = 0.07). **c** Change in plasma HDL-cholesterol concentration (r = 0.53, P = 0.03). **d** Change in finger reactive hyperaemic index (r = 0.49 P = 0.04). **e** Change in Matsuda insulin sensitivity index (ISI) (r = 0.52, P = 0.03). **f** Change in glucose area under the curve (AUC_0–120_) during oral glucose tolerance test (r = −0.61, P = 0.009)

Post weight loss change in exogenous insulin clearance (n = 17) correlated positively with changes in HDL-cholesterol (r = 0.53 P = 0.03), cardiac output (r = 0.44, P = 0.08), M (r = 0.45, P = 0.07) and inversely with changes in fasting calf vascular resistance (r = −0.63 P = 0.007), calf vascular resistance during OGTT (AUC_0-120_) (r = −0.58, P = 0.01) and *hs*-CRP (r = −0.49, P = 0.05) (Fig. 4). Post weight loss change in fasting C-peptide to insulin ratio correlated positively with changes in Matsuda ISI (r = 0.52, P = 0.03) and finger reactive hyperaemic response (r = 0.49, P = 0.04) and inversely with changes in glucose AUC_0-120_ (r = −0.61, P = 0.009) and HOMA-IR (r = −0.53, P = 0.03) (Fig. 4). Changes in anthropometrics, sympathetic neural parameters (arterial norepinephrine concentration, norepinephrine spillover rate and MSNA), creatinine clearance, and urinary sodium and potassium excretion did not correlate with change in exogenous insulin clearance or hepatic insulin extraction.

Stepwise regression analyses are summarized in Table 3. Improvement in exogenous insulin clearance after weight loss intervention was independently predicted by reduction in fasting calf vascular resistance and increase in HDL-cholesterol, which together explained 55 % of the variance. Improvement in hepatic insulin extraction after weight loss was independently predicted by enhanced glucose tolerance (reduced glucose AUC_0–120_) and finger reactive hyperaemic response, which together explained 64 % of the variance. In turn, change in fasting calf vascular resistance was independently predicted by improvement in insulin clearance and reduction in *hs*-CRP; enhancement in vasodilatory response during OGTT was predicted by improvement in insulin sensitivity; whilst change in the reactive hyperaemic response was inversely and independently predicted by change in MAP. Regarding SNS parameters, change in arterial norepinephrine concentration post weight loss was the strongest independent predictor of change in MAP, whilst reduction in MSNA was a function of improved baroreflex sensitivity (Table 3).

**Table 3:** Stepwise regression analyses of changes in key study parameters in the HCD group (n = 17)

Dependent variables	Step	Predictor variables	Std.coeff	r^2^	P	β
Insulin clearance						
Δ Whole-body insulin clearance (mL m^2^ min^−1^)	1	Δ Calf vascular resistance (units)	−0.53	0.40	0.01	0.94
	2	Δ HDL-cholesterol (mmol/L)	0.40	0.55	0.05	
Δ Fasting C-peptide to insulin ratio	1	Δ Glucose AUC_0-120_ (mmol/L/min)	−0.63	0.38	0.002	0.98
	2	Δ Finger reactive hyperaemic index	0.51	0.64	0.006	
Vascular						
Δ Fasting calf vascular resistance (units)	1	Δ Insulin clearance (mL m^2^ min^−1^)	−0.55	0.40	0.01	0.95
	2	Δ Log high sensitivity CRP (mg/mL)	0.40	0.55	0.047	
Δ Calf vascular resistance during OGTT (AUC_0–120_) (units)	1	Δ Fasting C-peptide (pmol/L)	0.63	0.48	<0.001	0.99
	2	Δ M/I (mg kg FFM min^−1^ mU/L × 100)	−0.47	0.69	0.008	
Δ Finger reactive hyperaemic index	1	Δ Mean arterial pressure (mmHg)	−0.57	0.33	0.02	0.69
Δ Mean arterial pressure (mmHg)	1	Δ Log arterial norepinephrine (pg/mL)	0.53	0.28	0.03	0.59
Δ MSNA (bursts/100hb)	1	Δ Cardiac baroreflex sensitivity (ms/mmHg)	−0.71	0.36	0.005	0.92
	2	Δ Body weight (kg)	−0.51	0.61	0.03	

Regression analyses were adjusted for age (years) and change in body weight (kg). β is the power of the test performed with α = 0.05

## Discussion

Hyperinsulinemia is considered to be a core component in the pathophysiology of obesity-related comorbidities [1, 2]. The focus of this study was insulin clearance, a key determinant of fasting and postprandial plasma insulin levels, which is known to be reduced in obesity particularly when features of the metabolic syndrome are present [5]. We simultaneously evaluated changes in sympathetic neural parameters, vascular and metabolic function, and anthropometrics following a HCD in order to ascertain the drivers of improvement in insulin clearance during lifestyle intervention. There are three main findings from our study. First, that weight loss of −8.3 % of baseline body weight elicited significant improvement in whole-body insulin clearance (by 12 %) and a non-significant increase in fasting hepatic insulin extraction (by 17 %) within our cohort. Second, that although vascular responses to weight loss were heterogeneous, reduction in calf vascular resistance was the strongest independent predictor of improvement in exogenous insulin clearance, whilst improvements in endothelial function (quantified as the finger reactive hyperaemic response) and glucose tolerance predicted improvement in endogenous insulin clearance. Third, that the sympathoinhibition attained following HCD was modulated by baseline insulin status and did not correlate directly with changes in insulin clearance.

Relatively few studies to date have examined the effects of caloric restriction on insulin clearance in obese adults and these have pertained to changes in endogenous, but not exogenous insulin clearance [3, 15, 37, 38]. Bosello et al. reported an average increase of 63 % in fasting C-peptide to insulin ratio after 3-weeks of a very low calorie diet (VLCD, 320 kcal/day) which resulted in a 6.3 % body weight loss [37]. Polonsky et al. studied changes in 24-h endogenous insulin clearance in different metabolic sub-groups following a VLCD (600 kcal/day) over a 6-week period [38]. Weight loss of 16–19 % in body weight translated to improvements of 30–50 % in insulin clearance, with greatest benefits in type 2 diabetic subjects. In another study 9.8 % weight loss over 9 weeks increased endogenous insulin clearance in response to graded glucose infusion by 42 % [3]. In our study, improvement in endogenous insulin clearance averaged 17 % which is smaller in magnitude than the aforementioned studies. It is likely that these differences may be explained by the more moderate caloric restriction and longer intervention period utilized in our study. Both the level of negative energy balance and weight loss per se are known to influence changes in insulin sensitivity, metabolic and autonomic parameters [39, 40].

The salient finding of our study is that improvement in vascular parameters (reduction in calf vascular resistance and increase in finger reactive hyperaemic index), independent of weight changes, delineated those subjects who gained a benefit in insulin clearance following HCD from those who did not. Moreover, this relationship was bidirectional as enhanced insulin clearance post weight loss independently predicted reduction in calf vascular resistance. These data concur with previously published studies showing that hypertension is associated with decreased insulin clearance [10, 12] and that administration of the α_1_-adrenergic agonist phenylephrine during hyperinsulinemic euglycemic clamp acutely diminishes insulin clearance in healthy volunteers [19]. Changes in peripheral vascular tone may impact on insulin clearance directly or indirectly via effects on insulin sensitivity. Impaired transcapillary transport of insulin caused by either decreased blood flow or permeability, or decreased capacity to vasodilate, could hinder the efflux of insulin from the intravascular space, hence decreasing insulin clearance [10, 20, 41], as well as impacting on insulin action [41, 42]. Our findings support the notion that insulin clearance and sensitivity are closely aligned [5–7] as improvement in Matsuda ISI and clamp derived glucose utilization post weight loss were positively associated with both measures of insulin clearance, and suggest that vascular function may be a common pathophysiological mediator of this association.

Our data underscore the potential importance of endothelial function in relation to insulin clearance at both the microvascular level and in skeletal muscle vasculature. Post ischemic finger reactive hyperaemia is a complex response, reflecting changes in digital blood flow and digital microvessel dilation, processes that depend on nitric oxide synthesis [35, 43]. Similarly, the vasodilatory response to endogenous insulin in skeletal muscle vasculature during OGTT is mediated by insulin-stimulated production of nitric oxide [44]. In our study, post weight loss improvement in these vascular parameters related to enhanced insulin clearance and insulin sensitivity and reduction in MAP and inflammatory milieu (plasma *hs*-CRP concentration). Other factors that may have contributed to improvement in endothelial parameters include weight loss and/or DASH diet induced changes in lipid profile (reduced plasma triglyceride and LDL-cholesterol and increased HDL-cholesterol concentrations) and reduction in glycemia [29, 45, 46]. Enhanced plasma nitrite level in response to reactive hyperaemia, has been reported in obese hypertensive subjects after 2-week consumption of a DASH eating plan, and suggest an upregulation of nitric oxide bioavailability [47]. Our finding of enhanced vasodilatory response to OGTT following HCD is relevant to postprandial glucose disposal and cardiovascular risk and concurs with other published data [48]. It is notable that the Insulin Resistance Atherosclerosis Study, which followed 748 non-diabetic participants over 5-years to examine prospective changes in insulin clearance, observed that higher systolic blood pressure, plasma triglycerides and waist circumference at baseline were associated with future decline of insulin clearance, whereas higher HDL-cholesterol level predicted an increase in insulin clearance [12]. These findings likely reflect the convergence of metabolic syndrome components and insulin resistance on endothelial function and support our findings regarding the benefits of weight loss. It merits emphasis, that vascular responses to weight loss were variable within our cohort. One explanation for this is that baseline differences in insulin status influenced the degree of sympathoinhibition attained independent of weight change. Insulin status has also been shown to affect the benefits of weight loss on flow-mediated vasodilation with favourable changes being manifest only in hyperinsulinemic individuals [36].

SNS activation promotes vasoconstriction, vascular structural changes, impaired endothelial function and capillary rarefaction and has been linked to the development of insulin resistance in both prospective population [49] and human experimental studies [21]. The level of sympathoinhibition attained in the present study was smaller than what we have previously reported in other cohorts with similar weight loss [50], and correlated with improvements in MAP, but not insulin clearance. Our subgroup analysis highlights that change in plasma insulin levels is a key determinant of the sympathoinhibitory response following weight loss [26].

The strengths of our study are the detailed characterization of study participants and the use of gold standard methodology to evaluate insulin sensitivity and clearance and SNS activity. The limitations of our study include the relatively small sample size and non-randomized study design. Also our study does not differentiate between the effects of negative energy balance and weight loss per se. Finally, the role of other unmeasured factors, for instance adiponectin, which may have impacted on endothelial function and insulin clearance, merits further investigation [51].

## Conclusions

Our study demonstrates a bidirectional inter-relationship between changes in insulin clearance and vascular endothelial function during the course of a weight loss program. Improvement in risk factors associated with the metabolic syndrome, namely insulin resistance, hyperglycemia, proinflammatory state, blood pressure and HDL-cholesterol may underlie the vascular benefit and enhancement in insulin clearance.

## Abbreviations

AUC_0–120_: area under the curve during oral glucose tolerance test (0–120 min)
BMI: body mass index
DASH: dietary approaches to stop hypertension
HCD: hypocaloric diet
Hs-CRP: high sensitivity C-reactive protein
HOMA-IR: homeostasis model assessment of insulin resistance
M: steady state glucose utilization during euglycemic clamp
M/I: steady state glucose utilization during euglycemic clamp, adjusted for steady-state plasma insulin concentration
MAP: mean arterial pressure
Matsuda ISI: Matsuda insulin sensitivity index
MSNA: muscle sympathetic nervous activity
OGTT: oral glucose tolerance test
PAT: peripheral arterial tonometry
SNS: sympathetic nervous system
VLCD: very low calorie diet
